# Acceptance and willingness-to-pay for oocyte cryopreservation in medical versus age-related fertility preservation scenarios among Swedish female university students

**DOI:** 10.1038/s41598-023-32538-z

**Published:** 2023-04-01

**Authors:** Pietro Gambadauro, Emma Bränn, Gergö Hadlaczky

**Affiliations:** 1 Department of Women’s and Children’s Health, Uppsala University, 751 85 Uppsala, Sweden; 2 Department of Learning, Informatics, Management and Ethics, Karolinska Institutet, 171 77 Stockholm, Sweden; 3 Centre for Health Economics, Informatics and Health Services Research, Stockholm Health Care Services, 171 77 Stockholm, Sweden; 4 Res Medica Sweden, 753 15 Uppsala, Sweden; 5 Institute of Environmental Medicine, Karolinska Institutet, 171 77 Stockholm, Sweden

**Keywords:** Health care, Infertility

## Abstract

Oocytes can be effectively cryopreserved and stored for future use in in-vitro fertilisation. Oocyte cryopreservation (OC) can therefore mitigate different threats to female fertility, but attitudes and policies often seem more favourable in medical rather than age-related fertility preservation scenarios. The value of OC for potential candidates may be perceived differently depending on the indications, although relevant empirical data are lacking. An adequately powered sample of Swedish female university students (n = 270; median age 25; range 19–35) were randomly delivered a medical (n = 130) or age-related (n = 140) fertility preservation scenario within an online survey. Sociodemographic factors, reproductive experiences, and awareness about OC were not significantly different between the groups. Differences in four outcomes were studied: proportions of respondents (1) positive to the use of OC, (2) positive to public funding for OC, or (3) open to considering OC; and (4) willingness-to-pay (WTP) for OC, measured in thousand Swedish krona (K SEK) through contingent valuation. There were no significant differences in the proportions of respondents positive to the use of OC (medical: 96%; age-related: 93%) or open to consider it (medical: 90%; age-related: 88%) in each scenario. However, public funding had significantly greater support in the medical scenario (85%) than in the age-related one (64%). The median WTP (45 K SEK ≈ 4.15 K EUR) approximated the current Swedish market price for a single elective cycle and was not significantly different between the scenarios (Cliff’s delta − 0.009; 95%CI − 0.146, 0.128). These findings suggest that it may be inappropriate to justify counselling and priority policies only on the assumption that fertility preservation with OC for medical indications is more beneficial to women than when the same technique is used for age-related reasons. However, it would be interesting to investigate further why public funding appears more debatable than the treatment itself.

## Introduction

Oocytes can be effectively cryopreserved and stored for future use in in-vitro fertilisation (IVF)^1^. Oocyte cryopreservation (OC) is therefore widely used in fertility preservation programs, for instance before gonadotoxic treatments, adnexal surgery, or sex-reassignment treatments^2^. Because of increased confidence regarding its safety and efficacy, OC is also offered to women who have reasons, other than medical, to delay their reproductive plans and are concerned about age-related fertility decline^1^.

Decision-making regarding fertility preservation with OC can be challenging. Even assuming known costs or risks, and live birth rates similar to those with fresh oocytes^3^, the future need or use of frozen oocytes for IVF is hypothetical. Besides, attitudes and policies regarding OC often seem more favourable in medical rather than age-related scenarios. OC is in both cases available in Sweden and many other countries^4,5^. However, medical indications are often prioritized within public healthcare whereas fertility preservation for age-related reasons is not and has even been banned in some countries^5^. Gynaecologists may be more likely to discuss OC with their patients in case of a cancer diagnosis rather than for age-related threats to future fertility^6^. In a previous population-based survey, most 30–39-year-old Swedish women approved OC regardless of the indication, but significantly more of them would consider it in case of medical rather than “social” indications^7^.

The rationale for these differences is not fully understood and debated^8–12^. The value of OC for potential candidates may be perceived differently depending on the indications, although this hypothesis lacks adequate empirical support from relevant groups. This study evaluated differences in attitudes and perceived value of OC as a method to preserve fertility for medical versus age-related reasons. We investigated whether more Swedish female university students approve and would consider OC in a medical rather than age-related scenario. We also investigated whether there are differences in the proportion of students approving public funding or in their willingness-to-pay in each scenario. As a secondary objective, the study investigated whether individual-level sociodemographic factors or reproductive experiences are associated with attitudes or willingness-to-pay for OC.

## Materials and methods

### Design and population

This study was conducted within the OCREA multidisciplinary research project on oocyte cryopreservation. A digital survey was developed in Swedish by researchers with expertise in reproductive medicine and science, experimental psychology, health economic evaluation, and survey research. The survey was pilot-tested for functionality and face validity, and a final version was delivered through the LimeSurvey online platform^13^. Female students attending Swedish universities and aged between 18 and 35 were eligible. Invitations were published via targeted ads on student noticeboards and social media (i.e., Facebook). Participation was voluntary and anonymous.

### Instruments

The survey had four sections including open and, predominantly, closed-ended questions (e.g., multiple-choice or agree/disagree). The first section screened for eligibility as female university student of age 18–35. The second and third sections addressed general sociodemographic factors, reproductive experiences as well as general questions related to OC and alternative strategies (i.e., oocyte donation or adoption). The fourth section investigated the study outcomes after providing general information about OC for fertility preservation. The participants were randomly presented with one of two hypothetical scenarios depicting a 35-year-old childless woman who is facing an increased risk of future infertility because of either a medical condition or social reasons to delayed reproductive plans (Supplementary Table S1). The scenario was assigned automatically by simple randomization. Three agree/disagree items (including four levels and a “can’t decide”/”don’t know” option) investigated agreement with (1) the use of OC (“the woman should be able to choose whether to undergo OC”), (2) public funding (“society should support the woman’s expenses to undergo OC”), and (3) openness towards undergoing OC in the scenario (“if you were that woman… would you consider undergoing OC?”). A subsequent set of questions investigated the respondents’ willingness-to-pay (WTP) out-of-pocket for their own hypothetical OC in the assigned scenario, introduced by the following text: “What would you be prepared to pay for egg freezing if you were the 35-year-old childless woman in the aforementioned situation”. The WTP captures the perceived individual benefit in monetary terms and was measured in thousands Swedish krona (K SEK) using contingent valuation and the dichotomous choice approach^14^. The respondents were instructed to consider total costs per person (i.e., including oocyte storage and, when needed, repeated OC cycles) but not future expenses for IVF or pregnancy-related care. They were first proposed to pay 90 K SEK (“To help you answer the question, we will present you different amounts in thousands of Swedish kronor. Would you pay [proposed sum]"), with two possible responses (yes or no). The initial sum was based on twice the approximate market price of OC in Sweden (45 K SEK, including medical fees and medication; ≈ 4.15 K EUR), as two stimulation-retrieval cycles are often needed for adequate oocyte yield^15^. In two follow-up levels, the proposed amounts were either increased (after a “yes” answer) or decreased (after a “no” answer) with one third of the initial sum (30 K). As a result, each respondent’s WTP fell within a specific range (i.e., 0 ≤ WTP < 30 K; 30 K ≤ WTP < 60 K; 60 K ≤ WTP < 90 K; 90 K ≤ WTP < 120 K; 120 K ≤ WTP < 150 K; WTP ≥ 150 K). To finally obtain a precise estimate, an open-ended question asked for the highest acceptable WTP (“What is the maximum amount that you would be prepared to pay”) within the assigned range (including the possibility of zero WTP in the lowest range), followed by a certainty calibration question.

### Variables

Three binary outcome variables described the attitudes towards OC as fertility preservation strategy. Affirmative answers (i.e., “totally/partially agree” or “yes, absolutely/maybe”) defined a positive attitude towards the general use OC, its public funding, and the hypothetic personal use in the allocated scenario. The WTP was treated as a continuous variable. Sociodemographic variables included age (categorized as ≤ 24, 25–29 or ≥ 30), country of birth (Sweden or other), region (major or other), field of university studies (Humanistic/Social, Science, Medical or Technical), employment (yes or no), monthly income (< 10 K, 10-19 K, 20-29 K, ≥ 30 K SEK), stable partner (yes or no). Three of the 21 Swedish regions were defined as major since they include the country’s major conurbations (Stockholm, Göteborg and Malmö). Reproductive experiences included previous pregnancy, live birth, and subfertility, addressed in dichotomous (yes or no) variables. Subfertility was defined as having sought medical assistance to get pregnant or having unsuccessfully attempted to conceive for at least one year. Indirect (experience of) subfertility was defined as acquaintance with someone having difficulties to get pregnant. Perceived knowledge regarding OC was self-rated from 1 to 5 (5 = best knowledge) before and after receiving information about OC, and final ratings were dichotomized as lower (< 3/5) or higher (≥ 3/5) knowledge. Interest for OC was captured by questions investigating thoughts and plans about the procedure. Attitudes towards oocyte donation and adoption were addressed in specific questions (i.e., “would you consider…?”) and open attitudes were defined by affirmative answers (“yes, absolutely” or “yes, maybe”).

### Sample size determination

According to a previous population-based survey, more Swedish women agree with the use of OC for medical rather than “social” indications, in general (94% vs. 70%) and for themselves (78% vs. 47%)^7^. Based on those findings, a sample of 128 respondents (i.e., 64 per scenario) allows to detect a statistically significant difference in the proportion of respondents accepting OC in a medical versus age-related scenario, with 95% power and alfa 0.05^16^. Although no published data are available regarding WTP differences among alternative OC indications, the same sample size is also sufficient to detect a significant difference in continuous outcomes (e.g., WTP) assuming a medium effect size (e.g., d = 0.50), with 80% power and alfa 0.05^16^. Allowing for 50% incomplete surveys, we aimed at recruiting a target sample of 256 subjects.

### Data analysis

Participants’ characteristics were descriptively summarized and compared between the two scenario groups, using contingency tables and Chi-squared or Fisher’s exact test (with expected cell frequencies < 5). For the main objectives, differences between the scenarios were studied with Chi-squared or Fisher’s exact test (categorical outcomes), and with Wilcoxon rank-sum test (WTP). For the secondary objectives, associations between covariates and outcomes were assessed with Chi-squared or Fisher’s exact test (categorical outcomes), and with Wilcoxon rank-sum or Kruskal–Wallis test (WTP). Moderator analyses studied sociodemographic and reproductive covariates as potential effect modifiers of the association between scenarios and outcomes, using separate logistic regression analyses with interaction terms (for categorical outcomes) or stratified analyses (for WTP). Sensitivity analyses studied WTP differences between the scenarios in five subsets, obtained respectively after excluding respondents (A) with lower knowledge of OC, (B) not open to considering OC, (C) uncertain about the given WTP, or (D) providing outlier WTP values. The results of these analyses were plotted as medians with interquartile ranges (IQR), and as effect sizes (Cliff’s delta) with 95% confidence intervals (CI). An attrition analysis was conducted to compare characteristics of randomised respondents who did or did not complete the study. The statistical analyses were performed in R^17^ with RStudio^18^ for macOS (ver. 2022.07.1) and significance was defined by *p* < 0.05.

### Ethical approval

The study was granted exemption from ethical approval by the Swedish Ethical Review Authority (Ref. nr. 2021-05414-01, 3rd Nov 2021). Data collection was anonymous and compliant to EU General Data Protection Regulation (GDPR). Written information was delivered in advance, and informed consent was obtained from all participants before inclusion.

## Results

**Figure 1:**
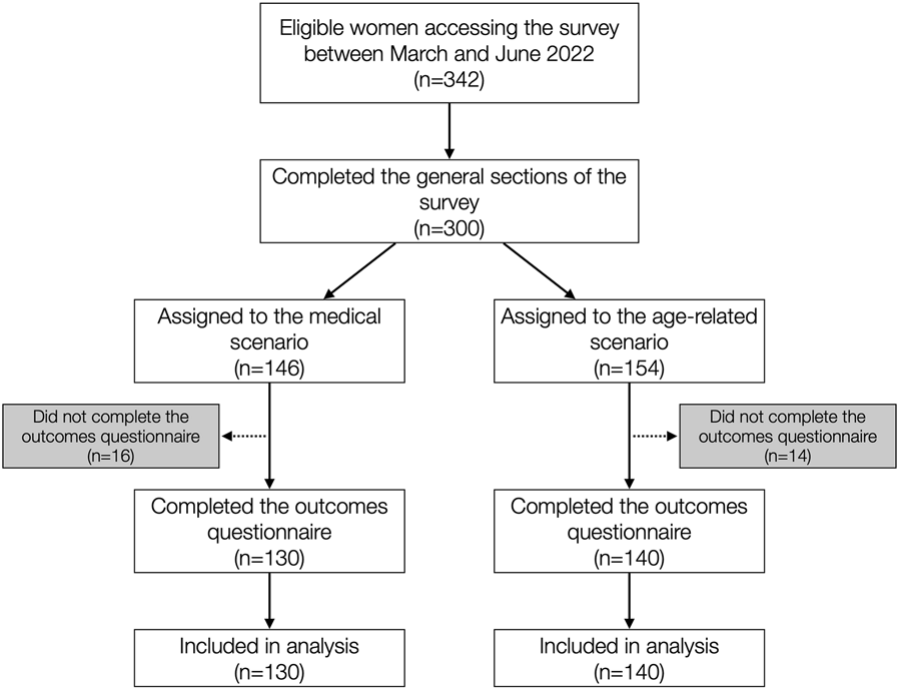
Study flowchart.

Between March and June 2022, 342 eligible women accessed the survey and 300 were assigned to the medical (n = 146) or age-related (n = 154) scenario after completing the general sections (Fig. 1). Of those, 270 (90%) completed the outcomes questionnaire and were included in analysis (median age 25; range 19–35; medical scenario n = 130; age-related scenario n = 140). Ninety percent were born in Sweden and 60% lived in one of the three major regions. The distribution of other sociodemographic factors is presented in Table 1. The proportions of respondents with experience of pregnancies, live births, subfertility, or indirect subfertility were 25%, 13%, 9.4%, and 68%, respectively. All but two (99.3%) had previously heard of OC, 57% had thoughts about it and 9.3% planned it. Less than a quarter had low perceived knowledge about OC (< 3 on a 1–5 scale). The majority could consider alternative family building strategies such as oocyte donation (52%) and adoption (72%).

**﻿Table 1:** Characteristics of the study population.

Variables	Levels	Overall (N 270)^a^	OC scenario group		
			Medical (N 130)^a^	Age-related (N 140)^a^	*p*-value^b^
Socio-demographic factors					
Age	19–24	123 (46%)	60 (46%)	63 (45%)	0.87
	25–29	85 (31%)	39 (30%)	46 (33%)	
	30–35	62 (23%)	31 (24%)	31 (22%)	
Born in Sweden	No	26 (9.6%)	14 (11%)	12 (8.6%)	0.54
	Yes	244 (90%)	116 (89%)	128 (91%)	
Living in a major region	No	109 (40%)	49 (38%)	60 (43%)	0.39
	Yes	161 (60%)	81 (62%)	80 (57%)	
Study field	Humanistic/Social	104 (40%)	46 (38%)	58 (43%)	0.73
	Medical	94 (36%)	48 (39%)	46 (34%)	
	Scientific	26 (10%)	11 (9.0%)	15 (11%)	
	Technical	34 (13%)	17 (14%)	17 (12%)	
	*Missing*	*12*	*8*	*4*	
Employed	No	91 (35%)	47 (38%)	44 (32%)	0.35
	Yes	171 (65%)	78 (62%)	93 (68%)	
	*Missing*	*8*	*5*	*3*	
Monthly income (SEK)	< 10 K	85 (32%)	38 (30%)	47 (34%)	0.92
	10-19 K	119 (45%)	57 (46%)	62 (45%)	
	20-29 K	25 (9.5%)	13 (10%)	12 (8.6%)	
	≥ 30 K	35 (13%)	17 (14%)	18 (13%)	
	*Missing*	*6*	*5*	*1*	
Stable partner	No	96 (36%)	46 (36%)	50 (36%)	> 0.99
	Yes	171 (64%)	82 (64%)	89 (64%)	
	*Missing*	*3*	*2*	*1*	
Reproductive experiences					
Pregnancy	No	202 (75%)	93 (72%)	109 (78%)	0.27
	Yes	67 (25%)	36 (28%)	31 (22%)	
	*Missing*	*1*	*1*	*0*	
Live birth	No	229 (87%)	110 (86%)	119 (88%)	0.71
	Yes	35 (13%)	18 (14%)	17 (12%)	
	*Missing*	*6*	*2*	*4*	
Subfertility	No	242 (91%)	113 (88%)	129 (93%)	0.20
	Yes	25 (9.4%)	15 (12%)	10 (7.2%)	
	*Missing*	*3*	*2*	*1*	
Indirect subfertility	No	87 (32%)	44 (34%)	43 (31%)	0.58
	Yes	183 (68%)	86 (66%)	97 (69%)	
OC related factors					
Perceived knowledge on OC	Lower	58 (22%)	34 (27%)	24 (18%)	0.075
	Higher	207 (78%)	94 (73%)	113 (82%)	
	*Missing*	*5*	*2*	*3*	
Thoughts about OC	No	115 (43%)	55 (42%)	60 (43%)	0.93
	Yes	155 (57%)	75 (58%)	80 (57%)	
Open for oocyte donation	No	129 (48%)	61 (48%)	68 (49%)	0.88
	Yes	139 (52%)	67 (52%)	72 (51%)	
	*Missing*	*2*	*2*	*0*	
Open for adoption	No	74 (28%)	34 (27%)	40 (29%)	0.63
	Yes	189 (72%)	93 (73%)	96 (71%)	
	*Missing*	*7*	*3*	*4*	

OC, oocyte cryopreservation.

^a^N (%).

^b^Pearson’s Chi-squared test or Fisher’s exact test (with expected cell frequencies < 5).

Missing observations (n) are in italics.

**Figure 2:**
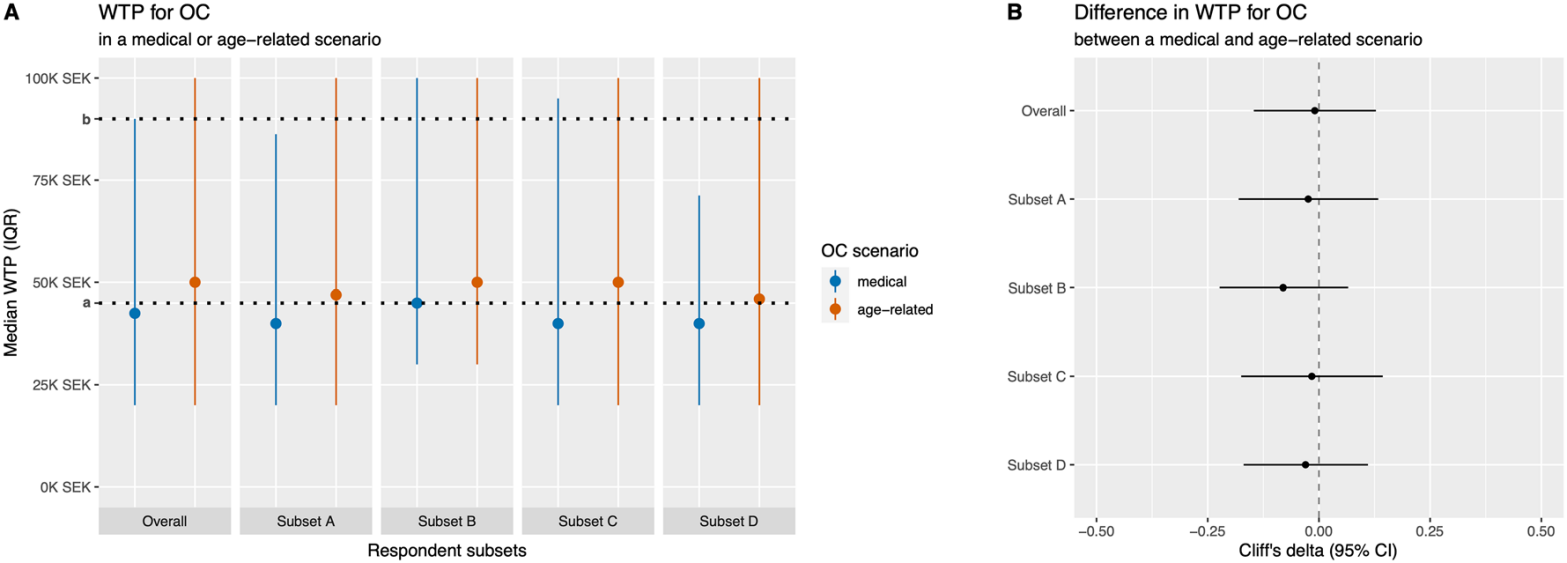
Willingness-to-pay (WTP) for oocyte cryopreservation (OC) in a medical or age-related fertility preservation scenario. Both plots display values obtained from the overall population and from subsets excluding respondents (Subset A) with lower knowledge about OC, (Subset B) not open to consider OC, (Subset C) uncertain about the given WTP, or (Subset D) providing outlier WTP values. (**A**) Comparison of median WTP, in thousand Swedish krona, in the two scenarios. The dotted lines represent the approximate market price for one (a = 45 K) or two (b = 90 K) elective OC procedures in Sweden. (**B**) Cliff’s delta values representing the difference between the probability that a randomly chosen WTP in the medical scenario is higher than one in the age-related scenario, and the probability of the reverse.

There were no significant differences in sociodemographic and reproductive factors between the two groups (Table 1). Most were in favour of OC for fertility preservation (94%), with no significant differences between the scenarios (Table 2). Many respondents were also positive about public funding for OC (74%), but the proportion was significantly higher in the medical scenario (Table 2). About 90% would consider undergoing OC in both scenarios (Table 2). The median WTP was 45 K SEK (IQR 80) with no significant differences between the scenarios (Cliff’s delta − 0.009; 95%CI − 0.146, 0.128; Table 2; Fig. 2).

**Table 2:** Attitudes and willingness-to-pay for oocyte cryopreservation (OC) in a medical or age-related fertility preservation scenario.

Outcome	Overall (N 270)^a^	OC scenario group		
		Medical (N 130)^a^	Age-related (N 140)^a^	*p*-value^b^
Positive to the use of OC	255 (94%)	125 (96%)	130 (93%)	0.24
Positive to public funding for OC	199 (74%)	110 (85%)	89 (64%)	< 0.001
Open to considering OC	240 (89%)	117 (90%)	123 (88%)	0.58
WTP^c^ for OC	45.0 (80.0)	42.5 (70.0)	50.0 (80.0)	0.89

^a^N (%) or median (IQR).

^b^Pearson’s Chi-squared test or Wilcoxon rank-sum test.

^c^Willingness-to-pay expressed in thousand Swedish krona (K SEK).

No sociodemographic or reproductive covariate significantly modified the above findings in moderator analyses. Similarly, sensitivity analyses found no significant differences in WTP between the scenarios in subsets of respondents (Fig. 2). No associations were found between sociodemographic or reproductive covariates and outcomes, except for greater support and higher WTP among those living in a major region (Table 3). The relationships between covariates and WTP were consistent in regression analyses accounting for the effect of the scenario group (Supplementary Table S2). Openness to oocyte donation or adoption significantly predicted a positive attitude towards considering OC as an option for oneself, regardless of the scenario (Table 3). Participants and respondents who dropped-out after randomization (n = 30) were not significantly different for background sociodemographic and reproductive characteristics (Supplementary Table S3).

**﻿Table 3:** Predictors of attitudes and willingness-to-pay for fertility preservation with oocyte cryopreservation (OC).

Variables	Levels	Positive to the use of OC		Positive to public funding for OC		Open to considering OC		WTP^a^ for OC	
		N (%)	*p*^b^	N (%)	*p*^b^	N (%)	*p*^b^	Median (IQR)	*p*^c^
Sociodemographic factors									
Age	19–24	117 (95%)	0.62	87 (71%)	0.48	112 (91%)	0.51	50.0 (77.5)	0.53
	25–29	81 (95%)		63 (74%)		73 (86%)		40.0 (55.0)	
	30–35	57 (92%)		49 (79%)		55 (89%)		40.0 (73.8)	
Born in Sweden	No	24 (92%)	0.64	18 (69%)	0.59	22 (85%)	0.51	60.0 (90.0)	0.14
	Yes	231 (95%)		181 (74%)		218 (89%)		42.5 (80.0)	
Living in a major region	No	98 (90%)	0.007	78 (72%)	0.51	94 (86%)	0.25	40.0 (40.0)	0.024
	Yes	157 (98%)		121 (75%)		146 (91%)		50.0 (80.0)	
Study field	Humanistic/Social	98 (94%)	0.26	79 (76%)	0.076	95 (91%)	0.29	40.0 (67.5)	0.38
	Medical	89 (95%)		73 (78%)		86 (91%)		45.0 (70.0)	
	Scientific	23 (88%)		14 (54%)		21 (81%)		37.5 (69.0)	
	Technical	34 (100%)		23 (68%)		29 (85%)		50.0 (68.8)	
Employed	No	88 (97%)	0.22	66 (73%)	0.92	81 (89%)	0.98	40.0 (52.5)	0.25
	Yes	159 (93%)		125 (73%)		152 (89%)		45.0 (70.0)	
Monthly income (SEK)	< 10 K	83 (98%)	0.30	59 (69%)	0.49	74 (87%)	0.92	45.0 (80.0)	0.86
	10-19 K	109 (92%)		88 (74%)		105 (88%)		50.0 (73.0)	
	20-29 K	24 (96%)		19 (76%)		23 (92%)		50.0 (70.0)	
	≥ 30 K	33 (94%)		29 (83%)		32 (91%)		40.0 (77.5)	
Stable partner	No	92 (96%)	0.44	66 (69%)	0.16	89 (93%)	0.13	40.0 (76.2)	0.77
	Yes	160 (94%)		131 (77%)		148 (87%)		45.0 (79.5)	
Reproductive experiences									
Pregnancy	No	193 (96%)	0.22	145 (72%)	0.24	179 (89%)	0.83	50.0 (80.0)	0.33
	Yes	61 (91%)		53 (79%)		60 (90%)		35.0 (35.0)	
Live birth	No	219 (96%)	0.10	168 (73%)	0.40	204 (89%)	> 0.99	45.0 (80.0)	0.27
	Yes	31 (89%)		28 (80%)		31 (89%)		35.0 (42.5)	
Subfertility	No	230 (95%)	0.16	180 (74%)	0.80	217 (90%)	0.18	45.0 (80.0)	0.79
	Yes	22 (88%)		18 (72%)		20 (80%)		35.0 (70.0)	
Indirect subfertility	No	84 (97%)	0.40	60 (69%)	0.22	78 (90%)	0.78	45.0 (70.0)	0.48
	Yes	171 (93%)		139 (76%)		162 (89%)		45.0 (79.5)	
OC related factors									
Perceived knowledge on OC	Lower	55 (95%)	> 0.99	43 (74%)	0.97	51 (88%)	0.84	50.0 (67.5)	0.41
	Higher	195 (94%)		153 (74%)		184 (89%)		40.0 (79.5)	
Thoughts about OC	No	104 (90%)	0.013	77 (67%)	0.030	88 (77%)	< 0.001	40.0 (79.5)	0.22
	Yes	151 (97%)		122 (79%)		152 (98%)		45.0 (73.0)	
Open for oocyte donation	No	117 (91%)	0.011	80 (62%)	< 0.001	100 (78%)	< 0.001	40.0 (60.0)	0.050
	Yes	136 (98%)		118 (85%)		138 (99%)		50.0 (70.0)	
Open for adoption	No	68 (92%)	0.37	50 (68%)	0.18	60 (81%)	0.016	46.0 (87.2)	0.92
	Yes	180 (95%)		143 (76%)		173 (92%)		45.0 (74.0)	

^a^Willingness-to-pay expressed in thousand Swedish krona (K SEK).

^b^Pearson’s chi-squared or Fisher’s exact test (with expected cell frequencies < 5).

^c^Wilcoxon rank-sum or Kruskal–Wallis test.

## Discussion

### Principal findings

Swedish female university students accept and would consider OC regardless of whether fertility preservation is pursued for medical or age-related reasons. In addition, the perceived value of OC for medical or age-related reasons is not significantly different, according to a willingness-to-pay analysis. However, although many students also approve public funding, significantly more do this for medical rather than age-related indications. These findings are consistent across sociodemographic groups and in sensitivity analyses among subsets of respondents.

### Strengths and limitations

The random comparison between the medical and age-related scenario is original and mitigates sources of response bias (e.g., order-effects, demand characteristics, social desirability, or fairness/reciprocity norms)^19,20^. The perceived value of OC was captured in a standardised and structured manner through a willingness-to-pay analysis^14^. The final sample exceeded the requirements of a predetermined sample size based on power analysis, and the rate of missing values was very low (Table 1). The selected population provides a narrow but relevant perspective, as 18–35-year-old female university students arguably are representative of potential candidates and have adequate preunderstanding. Besides, individual level data on several sociodemographic factors and reproductive experiences were considered in moderator analyses, and the robustness of the WTP comparison was assessed in sensitivity analyses.

No sampling strategy is free from bias, and this study may have selectively recruited women with strong positive or negative attitudes to OC. However, the large observed support for OC is validated by previous Swedish population-based findings^7^; the proportion of respondents from major regions (60%) is consistent with official demographics, since 56% of Swedish women aged 18–35 live in one of those^21^; the contents of the scenarios and outcomes questionnaire were only available after randomization and no differences were found between randomized respondents who did and did not complete the study. Another limitation is that two standardized scenarios were used, while a wider range of possibilities make cases different from each other in real-life contexts. Attitudes towards OC applications are complex^22–25^ and potentially influenced by details not addressed in the study scenarios. As regards the determination of the WTP, it can be considered that a first fixed amount in the dichotomous approach may cause starting point bias, although this applies to both scenario groups and should therefore not affect the comparative analysis. Finally, the survey was delivered in Swedish and therefore the results may only concern students who live more permanently in the country.

### Interpretation

OC’s popularity for fertility preservation is increasing despite debates regarding its applications. Decision-making is challenging because of uncertainty surrounding the utilization rates of frozen oocytes and thus the effectiveness of the procedure. This knowledge gap hinders comprehensive ethical and cost-effectiveness analyses, which are otherwise powerful decision-making tools. Nevertheless, fertility preservation with OC is accepted in accordance with the ethical principles of autonomy (e.g. individual reproductive choices), beneficence (e.g. avoiding infertility), non-maleficence (e.g. acceptable health risks), and justice (i.e. fairness from a societal perspective)^26^. In Sweden, for example, young women risking infertility due to medical reasons are offered OC in public healthcare^2^. OC for age-related reasons is also available in Sweden and many other countries, although not as a publicly-funded option^5^. Assuming that, in terms of outcomes, safety and costs, OC is non-inferior when performed for age-related rather than medical reasons, its value to individuals and society may be perceived differently depending on the indication. This would be consistent with moral arguments that OC for medical conditions implies a “need” whereas OC for age-related reasons is the result of a “choice” among other possible alternatives^8^. However, these arguments are controversial^8,10–12^ and poorly supported by empirical data concerning the perceived benefits of OC in different scenarios.

Previous surveys have studied attitudes towards OC for age-related concerns, although very few have compared them with attitudes towards OC when fertility is threatened by a medical condition or treatment. Planned OC receives variable support among young adult women internationally^7,22–25,27–32^. In this regard, Swedish female university students seem to be more open to this application of OC than peers from China (46%)^32^, Italy (19.5%)^31^, Singapore (48.9%, only medical students)^30^ or the US (71%, only medical students)^25^. Increasing awareness and knowledge may explain greater support for OC in more recent samples^25^, especially among those from major urban environments^7,32^ where facilities for OC are also usually available. In our study, openness to considering OC was also more likely among those positive for egg donation or adoption. This may seem counterintuitive since interest in OC as a preventive intervention can be lower if other strategies (e.g., egg donation or adoption) are perceived as viable alternatives. Probably, women who value parenting more are also more likely to consider alternative forms of family building.

Significant differences in attitudes towards medical versus age-related applications of OC are suggested by previous surveys. In a survey among Irish women, 72% would consider OC for fertility preservation but 25% only approved it for medical reasons as before cancer treatment^29^. In an internet-based study recruiting women from Denmark and the UK, most were aware and in favour of OC for fertility preservation^22^. However, more women found OC unacceptable in case of “social” (14–21%) rather than medical reasons (1–3%)^22^. More Canadian women participating in an online survey could approve the use of OC (91.4%) or consider it for themselves (81.6%) before cancer treatment rather than in case of not being “ready to have a child” (66.6% and 52.8%, respectively)^24^. A previous Swedish survey reported positive attitudes towards OC among most women (average age 34.4 ± 2.8; age range 30–39), regardless of the indication^7^. However, significantly more women supported OC in medical rather than “social” scenarios (94% versus 70%)^7^. The proportion of women who would consider undergoing OC was also significantly higher for medical (78%) versus “social” (47%) reasons^7^.

In contrast, our results show no significant differences in attitudes toward general and personal use of OC, or in the WTP, in a medical or age-related scenario. One possible explanation concerns the timing and population of the different surveys. Awareness and acceptance for OC have likely increased through time and may be particularly high in specific populations, such as young adult university students. Another explanation is that our study investigated attitudes in relation to two scenarios that were almost identical except for the threat to fertility (medical versus purely age-related). More importantly, only one scenario was randomly delivered within each survey to counteract bias that can influence responses when different indications are evaluated side-by-side, as competing alternatives^19,20^. A further peculiarity of the study is that it sought insights into what “acceptance” or “openness” for OC mean to people, addressing attitudes towards public funding and WTP. This is important because acceptance could otherwise be identified with agreement with public funding, especially where publicly funded fertility care is available (e.g., Sweden), or with commodification, at the other extreme. Through different outcome questions, the study addressed social (e.g., choosing/paying for others) and personal (e.g., choosing/paying for oneself) perspectives on OC separately, although those can arguably influence each other e.g. when eliciting preferences for a group of people among whom one could find oneself (i.e., what Dolan et al. call “socially inclusive personal preferences”)^33^.

Indeed, public funding appeared less obvious than the use of OC itself in this study. Not all respondents agreed with it for medical reasons even if that is available in Sweden, and significantly less approved it in the age-related scenario. This is consistent with a previous Canadian survey where more respondents approved public funding for cancer patients (80.2%) than for women who are not “ready to have a child” (45.4%)^24^. Despite a liberal attitude, young Swedish women may therefore see OC as an individual choice guided by subjective values, particularly if for age-related reasons^8^. However, even the controversially named “social” cryopreservation has medically-motivated intentions (i.e., preventing or reducing the risk of future infertility), and may not unequivocally be regarded as an independent choice^8,9,11^. Besides, external funding for OC may be disapproved for being an unfair facilitator for convenience- or ambition-driven women or, at the other extreme, a coercive tool in a male-oriented society^10^. Those arguments have however fallacies^10^ and could apply independently of the existence of medical threats to fertility.

WTP estimates in our study were not significantly predicted by reported income, regardless of the scenario. It is beyond the scope of this study to provide an empirical explanation to this finding, but its interpretation should take into account the potential influence that fairness, efficiency or sustainability considerations may have on preferences in social insurance contexts such as the Swedish/European one, which could be further explored in qualitative studies^33^. Furthermore, based on recent Australian data, Keller et al. hypothesize that women may be willing to pay for fertility treatments regardless of their income level due to the peculiar value they place on parenthood, which would make these treatments different from other goods^34^. In addition to the above considerations, the interpretation of the overall WTP estimates in our study may also consider the ex-ante (i.e., hypothetical) nature of the scenarios as well as the peculiarities of a sample consisting of young university students, who often share a limited economy based on temporary student loans or part-time employments. However, it is worth noting that the average WTP approximated the current Swedish price for a single elective OC procedure and few participants matched or exceeded the cost of two procedures (Fig. 2). Milman et al.^28^ previously studied the WTP for elective OC in a representative sample of American women. In that study, the WTP among women who would consider OC was significantly lower than the estimated cost of the procedure, even when assuming a 40% chance of live birth following treatment (WTP 3.8 K USD ≈ 42 K SEK or 3.9 K EUR). Higher costs could be considered (10 K USD) but only with a guarantee of ≥ 50% chance of live birth^28^. Interestingly, according to a study based on a Markov model^35^, elective OC for 35-year-old women would be cost-effective assuming three stimulation/freezing cycles, a 61% utilization rate at age 40, and for a total cost (including hypothetical costs for IVF/ET and miscarriages) of almost 20 K EUR (≈ 216 K SEK) per additional live birth, compared to spontaneous conception attempts followed, when necessary, by conventional IVF. Overall, these findings suggest that, due to financial reasons, few of those interested will eventually undergo OC^24^, or that many may agree to pay for it but not to the extent necessary to maximize their chances of success (e.g., for one single cycle regardless of the number of stored oocytes).

## Conclusions

It may be inappropriate to justify counselling and priority policies only on the assumption that OC for medical indications is more beneficial to women than when the same technique is used for age-related reasons. However, it would be interesting to replicate this study in different settings or subpopulations, and to investigate further why public funding appears more debatable than the use of OC itself.

## Supplementary Information

Supplementary Information.

## Data Availability

The data underlying this article cannot be shared publicly for the privacy of individuals that participated in the study. The data will be shared on reasonable request to the corresponding author.
