# Supplementary material for: Acceptance and willingness-to-pay for oocyte cryopreservation in medical versus age-related fertility preservation scenarios among Swedish female university students
Source: Sci Rep. 2023 Apr 1;13:5325. doi: 10.1038/s41598-023-32538-z (PMC10067828; doi:10.1038/s41598-023-32538-z)
Supplement: Supplementary file 1 — Supplementary Information. [file 41598_2023_32538_MOESM1_ESM.pdf]

Supplementary Information

Acceptance and willingness-to-pay for oocyte cryopreservation in medical versus age-related fertility preservation scenarios among Swedish female university students

Pietro Gambadauro [1,2,3,4,*], Emma Bränn [5], Gergö Hadlaczky [2,3]

Supplementary Table S1. Scenarios used in the study [a]

**Medical scenario**	**Age-related scenario**
Now we ask you to imagine a childless 35-year-old woman who will…	
…undergo medical treatment due to illness. The treatment…	*...postpone childbirth until later in life for social reasons. Aging…*
…affects the egg cells and thus entails an increased future risk of not being able to become pregnant with own eggs.	

[a] Translated from Swedish

Supplementary Table S2. Relationships between predictors and willingness-to-pay for fertility preservation with oocyte cryopreservation (OC), adjusted for the effect of the scenario.

Predictor		*Estimate [a]*	*95% CI [b]*		*P value*
**Sociodemographic factors**					
Age	19-24	*Reference level*	-	-	-
	25-29	-9.82	-35.91	16.28	0.46
	30-35	-7.82	-36.63	20.99	0.59
Born in Sweden	No	*Reference level*	-	-	-
	Yes	-11.50	-49.63	26.63	0.55
Living in a major region	No	*Reference level*	-	-	-
	Yes	23.74	0.96	46.51	0.04
Study field	Humanistic/Social	*Reference level*	-	-	-
	Medical	23.05	-3.56	49.67	0.09
	Scientific	-0.62	-41.56	40.31	0.98
	Technical	21.85	-15.05	58.76	0.24
Employed	No	*Reference level*	-	-	-
	Yes	23.64	-0.41	47.70	0.05
Monthly income (SEK)	<10K	*Reference level*	-	-	-
	10-19K	-2.04	-28.59	24.51	0.88
	20-29K	2.16	-40.40	44.71	0.92
	≥30K	12.58	-24.97	50.13	0.51
Stable partner	No	*Reference level*	-	-	-
	Yes	-7.06	-30.69	16.56	0.56
**Reproductive experiences**					
Pregnancy	No	*Reference level*	-	-	-
	Yes	-6.59	-32.71	19.54	0.62
Live birth	No	*Reference level*	-	-	-
	Yes	-8.41	-42.26	25.43	0.62
Subfertility	No	*Reference level*	-	-	-
	Yes	9.92	-29.19	49.03	0.62
Indirect subfertility	No	*Reference level*	-	-	-
	Yes	0.00	-24.08	24.08	1.00
**OC related factors**					
Perceived knowledge on OC	Lower	*Reference level*	-	-	-
	Higher	2.07	-25.67	29.80	0.88
Thoughts about OC	No	*Reference level*	-	-	-
	Yes	9.25	-13.47	31.96	0.42
Open for oocyte donation	No	*Reference level*	-	-	-
	Yes	9.48	-12.29	31.24	0.39
Open for adoption	No	*Reference level*	-	-	-
	Yes	-23.34	-48.83	2.15	0.07

[a] Ordinary Least Squares (OLS) regression derived estimates of the association between each predictor and willingness-to-pay (dependent variable), accounting for the effect of the OC scenario group (i.e., medical/age-related).
[b] Confidence interval

Supplementary Table S3. Comparison between respondents who did and did not complete the study after randomisation.

Variables	Levels	Randomized (N 300) [a]	Drop-out after randomization		
			No (N 270) [a]	Yes (N 30) [a]	p-value [b]
**Socio-demographic factors**					
Age	19-24	139 (46%)	123 (46%)	16 (53%)	0.63
	25-29	92 (31%)	85 (31%)	7 (23%)	
	30-35	69 (23%)	62 (23%)	7 (23%)	
Born in Sweden	No	28 (9.4%)	26 (9.6%)	2 (6.9%)	>0.99
	Yes	271 (91%)	244 (90%)	27 (93%)	
	missing	*1*	*0*	*1*	
Living in a major region	No	122 (41%)	109 (40%)	13 (45%)	0.64
	Yes	177 (59%)	161 (60%)	16 (55%)	
	missing	*1*	*0*	*1*	
Study field	Humanistic/Social	115 (40%)	104 (40%)	11 (39%)	0.99
	Medical	105 (37%)	94 (36%)	11 (39%)	
	Scientific	29 (10%)	26 (10%)	3 (11%)	
	Technical	37 (13%)	34 (13%)	3 (11%)	
	missing	*14*	*12*	*2*	
Employed	No	103 (35%)	91 (35%)	12 (41%)	0.99
	Yes	188 (65%)	171 (65%)	17 (59%)	
	missing	*9*	*8*	*1*	
Monthly income (SEK)	<10K	95 (33%)	85 (32%)	10 (36%)	0.63
	10-19K	134 (46%)	119 (45%)	15 (54%)	
	20-29K	26 (8.9%)	25 (9.5%)	1 (3.6%)	
	≥30K	37 (13%)	35 (13%)	2 (7.1%)	
	missing	*8*	*6*	*2*	
Stable partner	No	107 (36%)	96 (36%)	11 (41%)	0.62
	Yes	187 (64%)	171 (64%)	16 (59%)	
	missing	*6*	*3*	*3*	
**Reproductive experiences**					
Pregnancy	No	226 (76%)	202 (75%)	24 (83%)	0.36
	Yes	72 (24%)	67 (25%)	5 (17%)	
	missing	*2*	*1*	*1*	
Live birth	No	253 (87%)	229 (87%)	24 (86%)	0.78
	Yes	39 (13%)	35 (13%)	4 (14%)	
	missing	*8*	*6*	*2*	
Subfertility	No	267 (90%)	242 (91%)	25 (86%)	0.51
	Yes	29 (9.8%)	25 (9.4%)	4 (14%)	
	missing	*4*	*3*	*1*	
Indirect subfertility	No	99 (33%)	87 (32%)	12 (40%)	0.39
	Yes	201 (67%)	183 (68%)	18 (60%)	
**OC related factors**					
Perceived knowledge on OC [c]	Low	159 (53%)	140 (52%)	19 (63%)	0.23
	High	141 (47%)	130 (48%)	11 (37%)	
Thoughts about OC	No	131 (44%)	115 (43%)	16 (53%)	0.26
	Yes	169 (56%)	155 (57%)	14 (47%)	
Open for oocyte donation	No	144 (49%)	129 (48%)	15 (54%)	0.58
	Yes	152 (51%)	139 (52%)	13 (46%)	
	missing	*4*	*2*	*2*	
Open for adoption	No	83 (28%)	74 (28%)	9 (31%)	0.74
	Yes	209 (72%)	189 (72%)	20 (69%)	
	missing	*8*	*7*	*1*	

[a] N (%)

[b] Pearson's Chi-squared test or Fisher's exact test (with expected cell frequencies <5)

[c] Prior to receiving information regarding OC, which was delivered in the last section of the survey to those who went on to complete the study.
